# Near, far, wherever you are: simulations on the dose efficiency of holographic and ptychographic coherent imaging^1^

**DOI:** 10.1107/S1600576720005816

**Published:** 2020-05-27

**Authors:** Ming Du, Doǧa Gürsoy, Chris Jacobsen

**Affiliations:** a Advanced Photon Source, Argonne National Laboratory, Argonne, IL 60439, USA; b Department of Electrical Engineering and Computer Science, Northwestern University, Evanston, IL 60208, USA; c Department of Physics and Astronomy, Northwestern University, Evanston, IL 60208, USA; d Chemistry of Life Processes Institute, Northwestern University, Evanston, IL 60208, USA

**Keywords:** X-ray microscopy, radiation dose, ptychography, holography

## Abstract

Different studies in X-ray microscopy have arrived at conflicting conclusions about the dose efficiency of imaging modes involving the recording of intensity distributions in the near (Fresnel regime) or far (Fraunhofer regime) field downstream of a specimen. A numerical study is presented on the dose efficiency of near-field holography, near-field ptychography and far-field ptychography, where ptychography involves multiple overlapping finite-sized illumination positions. Unlike what has been reported for coherent diffraction imaging, which involves recording a single far-field diffraction pattern, it is found that all three methods offer similar image quality when using the same fluence on the specimen, with far-field ptychography offering slightly better spatial resolution and a lower mean error. These results support the concept that (if the experiment and image reconstruction are done properly) the sample can be near or far; wherever you are, photon fluence on the specimen sets one limit to spatial resolution.

## Abstract 2

Different studies in X-ray microscopy have arrived at conflicting conclusions about the dose efficiency of near- and far-field imaging approaches. X-ray far-field ptychography is compared with near-field holography and near-field ptychography in computer simulations. All three methods are highly dose efficient, with far-field ptychography offering slightly better low-dose imaging characteristics.

## 1. Introduction   

X-ray microscopy provides a unique combination of short-wavelength radiation (with the potential for nanoscale imaging) with high penetration. However, X-rays ionize atoms, so radiation damage often sets a limit on the achievable resolution, especially when studying soft or biological materials (Sayre *et al.*, 1977*a* ▸; Kirz *et al.*, 1995 ▸). This becomes quite important as one seeks finer spatial resolution 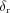, since for isotropic objects there is a tendency (Sayre *et al.*, 1977*b* ▸; Howells *et al.*, 2009 ▸) for the required number of photons per area incident on the specimen (the fluence 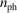) to obtain an image with sufficient signal-to-noise ratio to increase as 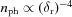. Since fluence leads directly to the absorbed radiation dose *D*, it is important to use low-fluence methods for high-resolution imaging.

One of the methods for low-fluence and low-dose X-ray imaging is to use phase contrast. That is because (Henke *et al.*, 1993 ▸; Du & Jacobsen, 2018 ▸) the phase shift imparted on an X-ray wavefront scales like 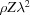, while beam absorption scales like 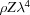, where ρ is the density, *Z* is the atomic number and λ is the wavelength. As a result, phase contrast often leads to reduced radiation dose for the same feature detectability, especially at shorter wavelengths (Schmahl & Rudolph, 1987 ▸).

While the phase of an X-ray wave cannot be measured directly, it can be inferred by mixing with a reference wave so that phase changes are encoded as intensity differences. This can be done using the Zernike method with X-ray zone plates (Schmahl *et al.*, 1994 ▸), or by using beam propagation. In near-field methods involving short propagation distances from the specimen to the detector, one or a few Fresnel fringes can be interpreted using approaches such as the transport of intensity (Paganin *et al.*, 2002 ▸), while at intermediate distances a large number of Fresnel fringes allow for in-line holographic reconstruction (Baez, 1952*a* ▸,*b* ▸) in an approach that is often referred to as near-field holography (NFH). One can improve reconstruction fidelity in NFH by combining information from holograms recorded at multiple distances (Cloetens *et al.*, 1999 ▸) or from multiple lateral illumination shifts (Stockmar *et al.*, 2013 ▸), where the latter approach is referred to as near-field ptychography (NFP). If instead the beam is allowed to propagate from the specimen to a detector at a distance that meets the far-field or Fraunhofer condition, X-ray images of phase objects can be recovered from coherent diffraction patterns with no wave mixing required (Sayre, 1980 ▸). This can be done in a single illumination approach now called coherent diffraction imaging or CDI (Miao *et al.*, 1999 ▸), where one uses finite-support iterative phase retrieval (Fienup, 1978 ▸). Alternatively, it can be done using multiple finite-sized overlappping coherent illumination spots in a method called far-field ptychography (FFP) (Hoppe, 1969*a* ▸,*b* ▸), where one again uses an iterative phase retrieval algorithm (Faulkner & Rodenburg, 2004 ▸) to obtain an image with a spatial resolution much finer than the size of the illumination spot (Rodenburg *et al.*, 2007 ▸).

Are there fundamental differences in photon exposure requirements depending on whether one mixes the specimen wave with a reference to get intensities, or measures the specimen wave diffraction intensities alone? One might think that by mixing a strong reference wave *R* with a weak specimen wave *S* one might have a multiplying effect due to the net intensity recording being 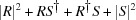, and indeed it has been suggested that NFH might be an especially dose-efficient imaging method (Bartels *et al.*, 2015 ▸; Hagemann & Salditt, 2017 ▸), though other simulation studies by some of the same researchers have found more of a dose equivalence with far-field diffraction (Jahn *et al.*, 2017 ▸). In fact, quantum noise is still limited by the specimen wave, leading to the following conclusion by Richard Henderson (1995 ▸) in the context of electron microscopy: ‘It can be shown that the intensity of a sharp diffraction spot containing a certain number *N* of diffracted quanta will be measured with the same accuracy (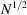) as would the amplitude (squared) of the corresponding Fourier component in the bright field phase contrast image that would result from interference of this scattered beam with the unscattered beam (Henderson, 1992 ▸). The diffraction pattern, if recorded at high enough spatial resolution, would therefore contain all the intensity information on Fourier components present in the image.’ This point is also addressed in Section 4.8.5 in the work of Jacobsen (2020 ▸). This leads us to expect that the reconstruction of a certain spatial frequency of the object should be equally accurate for far-field diffraction as it is for near-field phase contrast imaging, provided both use the same fluence 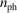 on a specimen pixel.

One could argue that the act of recovering phases from far-field diffraction patterns can introduce extra noise. Indeed, Henderson (1995 ▸) followed the comments above with this statement: ‘It [the diffraction pattern] would lack only the information concerning the phases of the Fourier components of the image which are of course lost. Thus, for the same exposure, holography should be equal to normal phase contrast in performance, and diffraction methods inferior because of the loss of the information on the phases of the Fourier components of the image.’ However, diffraction patterns *are* affected by the phase of Fourier components. Consider the example of a transverse shift of one subregion of a coherently illuminated object: the shift theorem of the Fourier transform makes it clear that one would change the phase of that subregion’s contribution to a specific point in the entire object’s complex diffraction amplitude. Therefore the intensity of the diffraction pattern produced by the object would undergo some redistribution (that is, the speckle pattern would change), showing that diffraction methods do indeed involve the encoding of phase. This is perhaps why a number of studies on iterative phase retrieval methods have indicated that the phase retrieval process seems not to add additional noise to the reconstructed image beyond that present in the diffraction pattern itself (Fienup, 1978 ▸; Williams *et al.*, 2007 ▸; Huang *et al.*, 2009 ▸; Schropp & Schroer, 2010 ▸; Godard *et al.*, 2012 ▸).

A slightly different approach to compare the signal-to-noise ratio for various imaging methods is to consider the strength of the signal scattered by a Gaussian-shaped feature characterized by a width 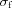, relative to the signal from the total illuminated area (the field of view or FOV) (Villanueva-Perez *et al.*, 2016 ▸). Unlike calculations that assume isotropic features and then calculate their contrast based on X-ray interaction properties (Sayre *et al.*, 1977*b* ▸; Howells *et al.*, 2009 ▸; Du & Jacobsen, 2018 ▸), this approach assumes that the feature scatters some number 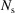 of photons for a given incident illumination (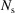 can be estimated; Shen *et al.*, 2004 ▸; Schropp & Schroer, 2010 ▸; Villanueva-Perez *et al.*, 2016 ▸). This approach has been used to calculate a signal-to-noise ratio (SNR) for propagation-based phase contrast microscopy [PM; equation (8) of Villanueva-Perez *et al.* (2016 ▸)] of
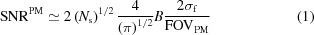
where 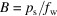 is the ratio of pixel size 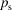 over source size 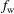 (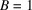 for coherent plane-wave illumination from a distant source in NFH). Analysis of coherent CDI [equation (9) of Villanueva-Perez *et al.* (2016 ▸)] yields an expression
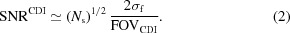
The FOV of CDI in equation (2) can be reinterpreted as the probe size in FFP. For a Gaussian probe, a reasonable way to define the probe size would be to consider a sharp-edged disc concentric with the probe, and having the same height (1) as the probe’s magnitude distribution. For the disc to have the same integral area as the probe, its diameter needs to be 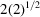 times the Gaussian probe’s standard deviation 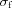. With this assumption, and expressing the FOV as 512 pixels for NFH and 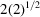 pixels for FFP, the SNR ratio between FFP and NFH becomes
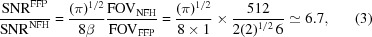
suggesting that FFP has a slight advantage over NFH.

In spite of equation (3), we hypothesize that the fluence 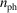 on the object sets the main limit on achievable resolution, rather than the use of near-field versus far-field imaging methods (assuming both methods are implemented in a way that allows a specific spatial resolution target to be reached). This hypothesis is supported by a previous simulation study of binary objects using propagation with different Fresnel numbers (Jahn *et al.*, 2017 ▸). Excluding the contact regime (where one loses sensitivity to phase contrast), this work concluded that near-field and far-field imaging methods require essentially the same critical photon fluence to reach the same level of reconstruction error. Nevertheless, this analysis was carried out using small objects with binary contrast and within rectangular supports, whereas we examine below the same irregularly sized objects with more continuous contrast that were used in a different near-field/far-field comparison (Hagemann & Salditt, 2017 ▸). In addition, both this binary object study (Jahn *et al.*, 2017 ▸) and other previous studies (Huang *et al.*, 2009 ▸; Hagemann & Salditt, 2017 ▸) used single diffraction patterns from finite-sized objects for far-field imaging. The reconstruction of complex objects from their single coherent diffraction patterns is not always straightforward, as one needs precise knowledge of the specimen’s support *S* (the subregion within which the object is restricted to lie; Fienup, 1987 ▸; Huang *et al.*, 2010 ▸). In addition, other experimental limitations like the loss of a significant subset of strong low-spatial-frequency intensity values due to the presence of beam stops can complicate object reconstruction (Thibault *et al.*, 2006 ▸; Huang *et al.*, 2010 ▸). These complications may have played a role in the simulation study noted earlier that showed that NFH yields superior images at the same fluence 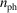 when compared with using standard CDI as a far-field imaging method (Hagemann & Salditt, 2017 ▸).

The problems noted above for standard CDI are greatly mitigated in FFP, where the finite coherent illumination spot provides several benefits. Ptychography allows one to accurately determine the equivalent of a finite support due not to the characteristics of the object but instead to the characteristics of the limited-size probe function, which can be recovered from the data. Object subregions that are present in the overlap between two probe positions provide a sort of holographic reference between the two resulting diffraction patterns (Bunk *et al.*, 2008 ▸). Finally, the spreading of the unmodulated probe function in the far field (due to its finite extent at the object’s plane) helps distribute intensities out of the central, zero-spatial-frequency pixel on the diffraction detector, especially when the probe is a convergent beam provided by the focus of a lens (Thibault *et al.*, 2008 ▸). Therefore while standard CDI often shows imperfections in image reconstruction beyond those provided by fluence, FFP can provide a method for a more robust comparison between the fluence requirements of near-field versus far-field coherent imaging methods. We have also shown good agreement between calculations and experimental FFP experiments for the fluence/resolution relationship when imaging integrated circuits (Deng, Hong *et al.*, 2017 ▸) and biological specimens (Deng, Vine *et al.*, 2017 ▸). It is for these reasons that we have carried out a simulation study comparing NFH not against CDI but against FFP as a far-field imaging method. We also include a comparison with NFP as a method that combines near-field recording, as in NFH, with multiple illumination positions, as in FFP.

## 2. Image reconstruction method   

In order to compare different imaging methods for non-binary objects, we have chosen to use the same optimization-based reconstruction method for the three imaging approaches, so as to reveal only the inherent differences between them. The work of Hagemann & Salditt (2017 ▸) used the relaxed averaged alternating reflections or RAAR algorithm (Luke, 2005 ▸) for reconstruction.

We have chosen to make use of the same simulated object that they used [shown in Fig. 2(*a*) of Hagemann & Salditt (2017 ▸)]. However, in our case we have chosen to use a more basic cost function minimization approach, in which one defines a forward model for how incident illumination interacts with a present guess of the object to produce a measurable intensity distribution, after which one seeks to adjust the object guess so as to minimize the difference between the result of this forward model and the actual measured intensity distribution (we refer to this difference as the cost function *C*). One can also include regularizers in this approach as will be described below. In order to efficiently minimize the cost function *C* for the three different imaging methods of NFH, FFP and NFP, we have chosen to use an automatic differentiation (AD) approach (Rall, 1981 ▸) so that we do not need to calculate gradients of *C* by hand for the two imaging methods and regularizers. The use of AD in CDI was suggested before powerful parallelized toolkits were widely available (Jurling & Fienup, 2014 ▸), but it has since been used for image reconstruction in FFP (Nashed *et al.*, 2017 ▸), in Bragg and near-field ptychography (Kandel *et al.*, 2019 ▸), and in NFH and FFP of thick specimens (Du *et al.*, 2020 ▸).

**Figure 1:**
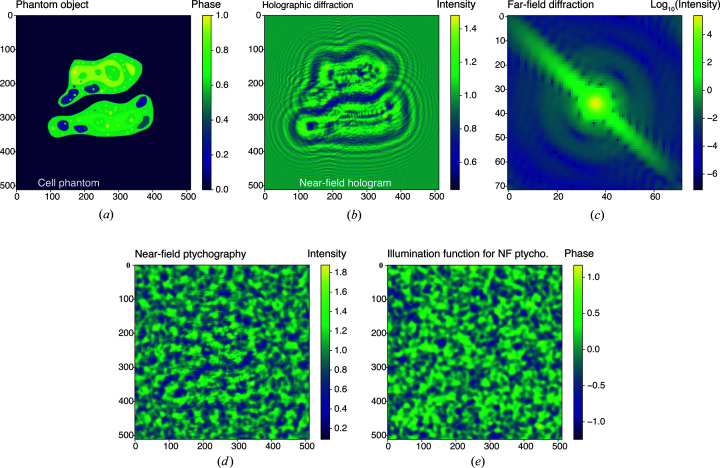
The 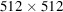 pixel phantom cell object used for our computational experiments (*a*). The object is the same pure-phase cell phantom used in a prior study (Hagemann & Salditt, 2017 ▸), so that one can compare directly with those results. The only difference is that we used the complex conjugate of the phantom so as to have positive rather than negative phase shifts, since X-ray phase is advanced rather than retarded in materials (Larsson *et al.*, 1924 ▸). (*b*) The simulated experimental intensities for NFH with propagation by a distance corresponding to a per-pixel Fresnel number [equation (6)] of 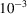. (*c*) One of the far-field diffraction patterns at the center of the object. In fact, a set of far-field diffraction intensity patterns were simulated for a series *k* of different illumination or probe positions across the sample, which is the type of data set one obtains in FFP. In (*d*) and (*e*), we show one of the NFP recordings of the object and the phase map of the illumination function used.

Our approach is to minimize the cost function *C* by adjusting the object function 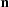 which contains the complex refractive index of the sample. For X-ray imaging, we used a 2D grid of the X-ray refractive index 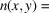 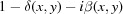 distribution multiplied by the projection object thickness 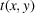 to yield an optical modulation of 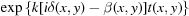 in the sign convention where forward propagation is 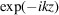. In our case, we used the same 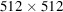 pixel pure-phase cell phantom [shown here in Fig. 1 ▸(*a*)] as was used in prior work (Hagemann & Salditt, 2017 ▸), with the modification of taking its complex conjugate so that it had positive rather than negative phase values since X-ray phase is advanced rather than retarded in materials (Larsson *et al.*, 1924 ▸). Within the 19.4% of the pixels that define the support *S* of the object, it produces an optical modulation 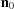 on the incident illumination with a mean phase of
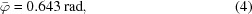
a variance of 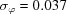
 rad and a bound of 0–1 rad (this object phase contrast is representative of what one might have in soft X-ray imaging; the contrast is usually lower in hard X-ray imaging). The cost function *C* is the mean squared difference between the modulus of the wave at the detector plane as predicted by the forward model 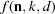 for the present guess 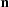 of the object, and the ‘measured’ intensity 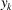 of
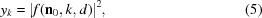
where *d* is the free-space propagation distance *z* in terms of a Fresnel number
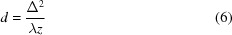
for an object pixel size Δ (so that far-field diffraction has 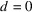). Fresnel propagation 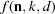 of the wavefield leaving the specimen to the detector plane was accomplished via convolution with a propagator function in the Fourier domain (Goodman, 2017 ▸). Poisson noise was incorporated in recorded intensity values 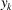 as will be described below. We then had a least-squares or LSQ cost function 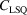 between the intensities one would expect from the present guess of the object versus the measured intensities 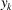 of
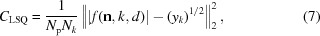
where 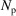 represents the number of pixels in the detector and 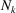 represents the number of illumination spots *k* (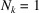 for the single, full-area illumination in holography).

The formulation of the cost function in equation (7) is straightforward: by minimizing the cost function, we update the object function 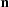 so that the Euclidean distance between the diffraction images generated by 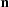 and the actual measurements is reduced. An LSQ cost function like this is more appropriate for images containing Gaussian noise, which is generally applicable at relatively high photon fluences (Cai *et al.*, 2017 ▸), but is unable to accurately account for the shot noise at low photon fluences. When the object is illuminated by a limited number of photons, the total probability of observing the entire set of experimental measurements given the object function 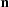 is better described by a Poisson distribution as
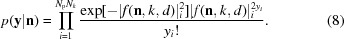
Equation (8) is also known as the Poisson likelihood function, and the true object function should be one that maximizes the likelihood. In practice, the negative logarithm of equation (8) is often taken, so that the maximization of a serial product can be turned into the more tractable problem of minimizing a sum. In this way, the Poisson cost function is written as
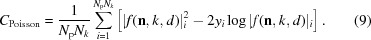
In NFP and FFP, the lack of scattering that takes place when the illuminating probe function is outside the object’s boundary means that it is quite natural for a reconstruction algorithm to seek solutions for such regions that are empty, even under conditions of limited illumination. To add a similar constraint *only* to NFH reconstructions, we added to the cost function of equation (7) a regularizer consisting of a finite-support mask *S*. This yields an update 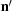 to the object of
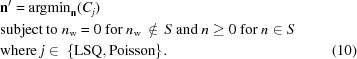
A finite-support constraint also suppresses the twin image in in-line holography (Liu & Scott, 1987 ▸). Due to the presence of information redundancy, FFP and NFP do not need a finite-support constraint.

With the forward model as described above, and the finite-support constraint added to NFH, we were able to obtain reconstructed images by minimization of the cost function *C*, using either the LSQ or the Poisson cost function. The partial derivative of *C* with regards to the elements of 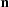 was calculated using AD as implemented as a cost function in *TensorFlow* (Abadi *et al.*, 2016 ▸), so that all three imaging types and both cost function types (LSQ and Poisson) could be treated in the same way simply by varying the Fresnel number *d*. The Adam optimizer (Kingma & Ba, 2015 ▸) in *TensorFlow* was used to update the object function using the calculated gradients.

## 3. Numerical experiments   

For direct comparison with prior work (Hagemann & Salditt, 2017 ▸), we used the same 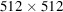 pixel simulated cell phantom phase object described above, and the same value of the Fresnel number [equation (6)] of 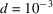 for NFH. This corresponds to *z* = 40.3 µm with Δ = 10 nm pixel size at a soft X-ray photon energy of 500 eV, or *z* = 807 µm at a hard X-ray photon energy of 10 keV. In the case of NFH, the object was padded by 256 pixels on each side before optical propagation was carried out in order to prevent fringe wrap-around due to the periodic array nature of discrete Fourier transforms. The finite-support mask is created by thresholding a low-pass-filtered version of the true object, so that the mask is about 9 pixels looser than the actual object boundary. For FFP, we assumed a probe function that was Gaussian in both magnitude and phase, with a standard deviation of 6 pixels and a phase that varied from 0 to 0.5 rad. The shift between probe positions was set to 5 pixels so that there was sufficient probe overlap at low fluence as is required for robust ptychographic reconstructions (Bunk *et al.*, 2008 ▸); this is discussed further in the supporting information. This led to a square scan grid with 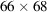 probe positions, and for each probe position a 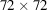 pixel subset of the object array was extracted before multiplication with the probe function and calculation of the resulting 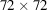 pixel diffraction pattern.

For our complementary study on NFP, the setup is assumed to be for a point-projection imaging, where a point source is used for illumination. The high spatial resolution of a point-projection microscope is achieved by the geometrical magnification effect of the spherical wave that the point source emits. As the Fresnel scaling theorem [Appendix *B* in the work of Paganin (2006 ▸)] indicates that this geometry is equivalent to plane-wave illumination with the sample–detector distance scaled by a certain factor, we can simulate the image-forming process simply using a plane wave as the probe function. Since NFP delivers better resolution when a diffuser is used to generate a structured illumination (Stockmar *et al.*, 2013 ▸), we created our incident illumination function as a wavefield with unity magnitude and random phase distribution. The phase map was generated by first creating a 768 × 768 array of Gaussian-distributed per-pixel random phases centered at 0 with σ = 0.3 rad; it was then spatially smoothed using a kernel with σ = 5 pixels. The phase of the illumination function (cropped to the same size as the final diffraction image) is shown in Fig. 1 ▸(*e*). A Fresnel number of 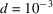 between the sample and detector, the same as the value used for NFH, is used in this case. After the sample-modulated wavefield was propagated to the detector plane, it was cropped down to 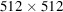 to remove fringe wrapping at the edges. Since each diffraction pattern in point-projection-based NFP has a much larger effective FOV (larger than the sample size) compared with FFP, a small number of scan spots suffice. If both the probe function and the object function contain *N* pixels, and so does each diffraction image, then it takes at least four diffraction patterns to solve both the object and the probe (Stockmar *et al.*, 2013 ▸). We therefore followed their choice of using 16 diffraction patterns distributed in a 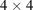 grid, through which the sample was translated across the entire 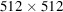 final FOV while being fully contained inside. This should provide sufficient data for a robust reconstruction provided that we use a known probe function.

X-ray microscopes use ionizing radiation, so it is important with many specimen types to limit the photon fluence 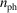 (average number of incident photons that hit each pixel containing the sample) and consequent radiation dose that the specimen receives. However, one must supply sufficient fluence in order to successfully image small low-contrast features. For phase contrast imaging of a non-absorbing low-contrast specimen with thickness 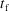 and phase-shifting part of the refractive index 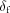 for feature-containing pixels and 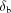 for background (feature-free) pixels, one can estimate that the fluence required to obtain an image with a signal-to-noise ratio of SNR is given by equation (39) from the work of Du & Jacobsen (2018 ▸), which we rewrite here as
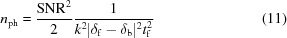
where 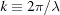 is the wavenumber. Since 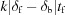 is the mean phase shift within the object compared with the object-free region, we can substitute this with 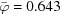
 rad from equation (4) and obtain an estimate that we require a fluence of
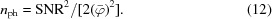
Given that the variance about the mean phase within the object was 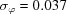
 rad, we would expect that an SNR of about 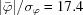 would begin to give very faithful low-noise representations of the true object, which corresponds to a fluence estimate of 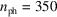 photons per pixel (with higher fluences giving increasing image fidelity).

**Figure 2:**
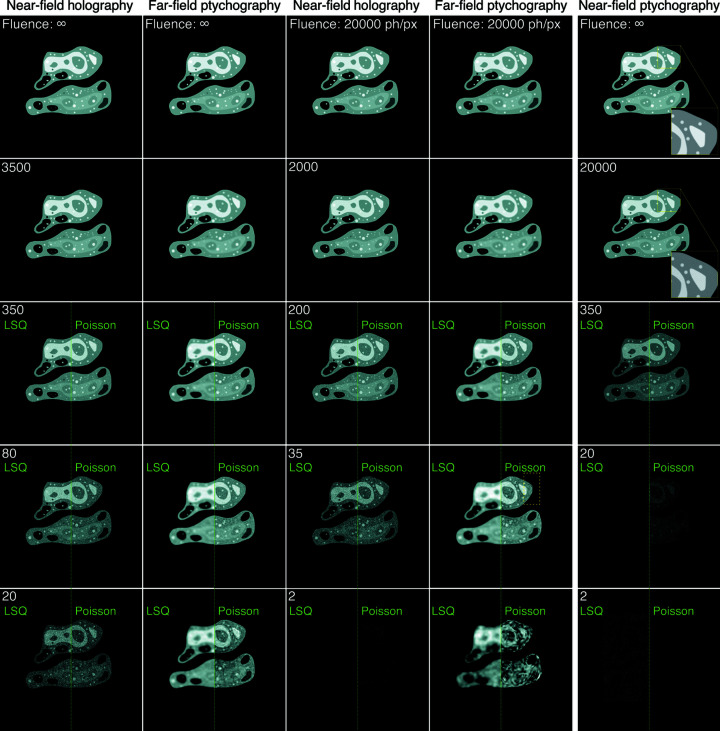
Reconstructed images of the cell phantom shown in Fig. 1 ▸(*a*) obtained for NFH, FFP and NFP at the photon fluences 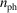 indicated. For a photon fluence higher than 350 photons per pixel, only results obtained using the LSQ cost function are shown; for fluences at or below that value, we show the reconstructions obtained using both the LSQ cost function [equation (7)] and the Poisson cost function [equation (9)]; these are placed side by side. At high photon fluence, both NFH and FFP yield high-quality images. However, their behaviors differ at low fluence. For NFH, the images gain a more salt-and-pepper appearance, as one would expect from low-photon statistics. The use of the Poisson noise model does not significantly improve the reconstruction quality. In FFP, the decrease in photons scattered beyond the illumination probe’s numerical aperture at low fluence means the images tend more and more towards the probe’s limit of spatial resolution. While the LSQ cost function gives blurry reconstructions at low photon dose, the results with the Poisson cost function preserve sharp features even at very low photon count, but instead show fringe-like artifacts. With NFP, using the Poisson cost function at low dose slightly improves the contrast in reconstructed images. However, both LSQ and Poisson results contain high-frequency artifacts that are eliminated only with noise-free diffraction data (see insets).

We therefore carried out simulations with values of 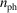 that bracketed a value of 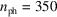 per pixel on an approximately logarithmic scale. Starting from the noise-free ‘recorded’ intensities 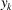 of equation (5), we incorporated Poisson noise to 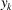 for a specified total fluence 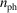 in photons per pixel on the specimen (to save computational time, NFP was tested on a subset of the photon fluence values used for NFH and FFP). Because we expect 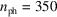 per pixel to be the nominal dividing line between ‘high-dose’ and ‘low-dose’ regimes, data sets with 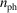 beyond that were reconstructed using the LSQ cost function which approximates photon noise using a quasi-Gaussian model that works well at high photon fluence. On the other hand, data with 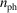 below 350 per pixel were reconstructed using both the LSQ and the Poisson cost functions. Two separate, independent random-noise data sets were generated for each experiment type, fluence and loss function type; reconstructed images from one of these two instances are shown in Fig. 2 ▸. This figure shows that both NFH and FFP yield high-quality reconstructions at high photon fluence. As the fluence decreases to 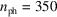 incident photons per pixel or less, the images begin to show a degradation in quality, but in different ways. In NFH, the images begin to take on a ‘salt-and-pepper’ or speckle-like noise appearance as one would expect in a direct coherent imaging experiment. Switching to the Poisson cost function does not help significantly with improving the quality. In FFP at low fluence, one will have relatively few photons scattered outside the numerical aperture of the probe function, so the image appears to show a loss of spatial resolution going towards the probe resolution but with less ‘salt-and-pepper’ noise appearance. At very low fluences in FFP, there are relatively few photons in the overlap regions between probe positions. If a sparser scan grid was used, one would start to see the scan grid artifacts that can arise due to insufficient probe overlap when using the LSQ cost function (Bunk *et al.*, 2008 ▸; Huang *et al.*, 2017 ▸). The 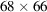 scan grid we used in this case is fine enough to suppress these artifacts, but a grid with doubled spot spacing could result in obvious grid artifacts, and in that case, the Poisson cost function turns out to be a better option (see supporting information, Fig. S1). The Poisson cost function is also able to give sharper boundaries of features compared with the LSQ cost function, especially for 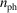 below 35 per pixel. Nevertheless, results of the Poisson cost function at relatively high photon fluences incorporate fringe-like artifacts, such as in the region marked by the yellow dashed box in the image with 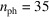 per pixel. Even when reconstructing noise-free data, this kind of artifact still exists, which proves that the Poisson cost function is not always a superior choice than LSQ and Gaussian cost functions. Another observation adding to this conclusion is that the Poisson cost function generally takes more iterations to converge, especially in the case of FFP.

For NFP, using a Poisson cost function improves the contrast of the reconstructed images to some extent. However, it was observed exclusively in NFP that almost all results obtained from noisy data, even with 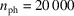 where NFH and FFP yield nearly identical results to the ground truth, contain high-frequency artifacts. When the input data are noise free, then NFP is able to reconstruct the image without artifacts, as shown in the insets in Fig. 2 ▸. The reduced performance at low fluence may be attributed to the ambiguity arising from noise-related uncertainty: although both a structured illumination and multiple diffraction images are used to provide information diversity, the presence of noise makes the solution non-unique. Tighter constraint usually leads to a better solution, which can be provided either by taking more diffraction images so that the uncertainty is reduced by larger sample volume or by using a finite-support constraint as in the case of NFH. However, a tight support constraint is not always easily determined and, furthermore, avoidance of the requirement of a finite-support constraint is in fact one of the motivations to use NFP.

**Figure 3:**
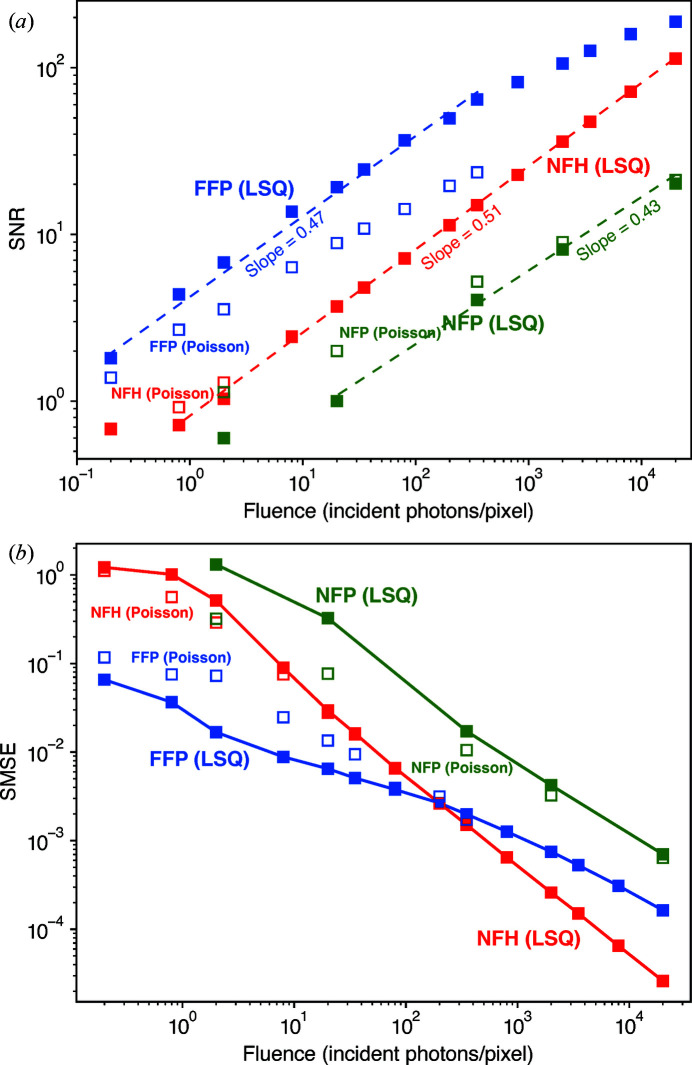
Whole-image metrics of image-reconstructed image quality as a function of fluence 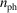. (*a*) The SNR as calculated using equation (14) for NFH, FFP and NFP, and using either the LSQ (solid squares) or Poisson cost function (open squares). The image correlation was calculated within the finite-support area of the object. At each photon fluence 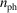 and for each cost function type, two separate instances of Poisson noise were generated and applied to the noise-free data set. The noisy data sets are then independently reconstructed and used for our correlation-based SNR calculation. The slope for the LSQ fitting curves is near 0.5 for all three techniques, indicating that the SNR increases roughly as 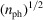, as one might expect. (*b*) The within-support mean squared error (SMSE) of equation (15), which shows improved performance at low fluences for FFP compared with NFH. NFP shows a larger SMSE for all photon fluences tested.

In order to better quantify the reconstruction quality, we now consider metrics one can obtain from noisy images. If one has two images 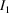 and 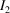 of the same object with two different instances of noise, one can calculate an overall image correlation coefficient *r* of (Bershad & Rockmore, 1974 ▸)
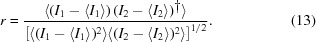
One can then use this correlation coefficient to calculate an overall image SNR (Frank & Al-Ali, 1975 ▸) of
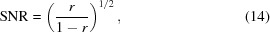
where the expression of equation (14) is correct for intensity images 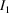 and 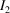, as confirmed by the as-expected scaling of 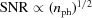 (Huang *et al.*, 2009 ▸). Although we do not use a finite-support constraint as part of the NFP and FFP reconstruction processes, for comparison with NFH we calculate *r* and SNR only within the finite-support region for all three imaging methods, leading to the result shown in Fig. 3 ▸(*a*). With the exception of the very lowest fluences in NFH and NFP, and NFP fluences above the 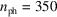 estimate given after equation (12) at which one expects to have achieved a high-fidelity reconstruction of the object, the SNR from all reconstruction methods shows a linear trend on this log–log plot with a slope of about 0.5 as expected for 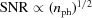. FFP shows the highest overall SNR, with NFH being second to it, and NFP the lowest. The high-frequency and uncorrelated artifacts in NFP results are clearly responsible for the method’s lower SNR. As one compares the results yielded by the two types of cost functions, it can be found that, while the SNR of NFH is slightly enhanced at 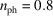 and 2 per pixel, the SNR of FFP reconstructions with the Poisson cost function is actually lower than those with LSQ, and the disparity increases at higher 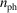. This observation seems to contradict the visual appearance of images in Fig. 2 ▸, where Poisson reconstructions give sharper feature boundaries under low-dose conditions. This could be explained by the fact that the method of calculating the SNR we have chosen measures the degree of correlation between two independently reconstructed images. If the images each contain correlated artifacts, the SNR is erroneously increased. When using the LSQ cost function to reconstruct FFP data, the loss of high-frequency information due to photon deficiency results in overall blurriness in the reconstructed images. In Poisson reconstructions, however, low photon fluence leads to localized fringe artifacts, which are heavily dependent on the initial guess. When 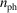 is sufficiently high that LSQ reconstructions are almost noise free, there is still a minor presence of the fringe artifacts in Poisson reconstructions. As the initial guess was created by Gaussian noise, the positions and numbers of the fringes can vary even for two reconstructions corresponding to the same 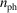. As a result, the SNR metric of equation (14) tends to interpret the artifacts in FFP reconstructions with the Poisson cost function as uncorrelated noise.

Since the phantom cell is a pure-phase object with a well defined support *S* (which was used in the NFH reconstruction to suppress the twin image), another whole-image metric we can use is the within-support mean squared error (SMSE) on the phase of
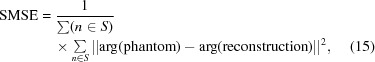
where *n* is a pixel index. This is the same 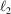-norm metric defined by equation (9) in prior work (Hagemann & Salditt, 2017 ▸). Our results for the SMSE for NFH, FFP and NFP are shown in Fig. 3 ▸(*b*). Hagemann & Salditt (2017 ▸) found that NFH gave a higher SMSE at fluences below about 100 quanta per pixel when compared with far-field CDI, but that holography then gave a lower SMSE at higher fluences. Here, we have found a very similar relation between NFH and FFP, with the SMSE cross-over also occurring near 100 photons per pixel. Other than that, we have again found that use of the Poisson cost function [equation (9)] gives slightly better results than LSQ [equation (7)] for NFH and NFP, but appears to result in larger SMSE for FFP, due to the more uncorrelated artifacts in FFP’s Poisson reconstructions.

**Figure 4:**
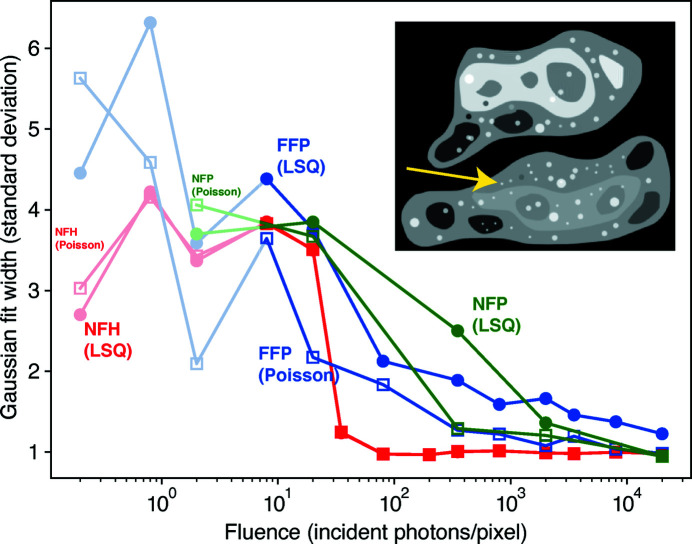
Standard deviation of the fitted Gaussian 2D profile for the small bright-spot-like feature pointed to by the yellow arrow. This is done for NFH, FFP and NFP. For photon fluences below 2 per pixel, the curves are greatly influenced by uncertainties, but meaningful results start to appear at higher photon fluences (with outliers for FFP at 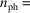 35 and 200 per pixel removed). The results agree with the visual appearance of the reconstructions shown in Fig. 2 ▸, where features in FFP reconstructions appear sharper when using the Poisson cost function at low photon fluence.

Although whole-image SNR measurements show that FFP slightly outperforms NFH (and largely outperforms NFP) at low photon fluence, they also seem to indicate improved results for FFP when using the LSQ cost function [equation (7)] instead of the Poisson cost function [equation (9)] at low fluence, which seems to contradict the visual appearance of the reconstructed images shown in Fig. 2 ▸. We therefore compared the performance of the NFH, FFP and NFP reconstructions for reconstructing a small, bright feature indicated by a yellow arrow in Fig. 4 ▸. For each reconstructed image, a Gaussian fit was carried out on this feature with a 2D symmetric profile, as shown in Fig. 4 ▸. An increase in the standard deviation of the Gaussian fitting function thus measures the blurriness of the reconstructed image, since a sharper feature will have a smaller standard deviation. At very low photon fluence, the overall resolution of the images is low, and the fitted standard deviation may suffer from significant uncertainty. With 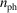 > 2 per pixel, the results start to show less fluctuation (outliers for FFP at 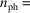 35 and 200 per pixel have been removed from the plot). For FFP, the plot of the Gaussian fit width better indicates the sharper features brought by the Poisson cost function, as the Gaussian spread of the feature in Poisson ptychography is smaller than that in LSQ ptychography. This agrees with visual perception of the results shown in Fig. 2 ▸.

**Figure 5:**
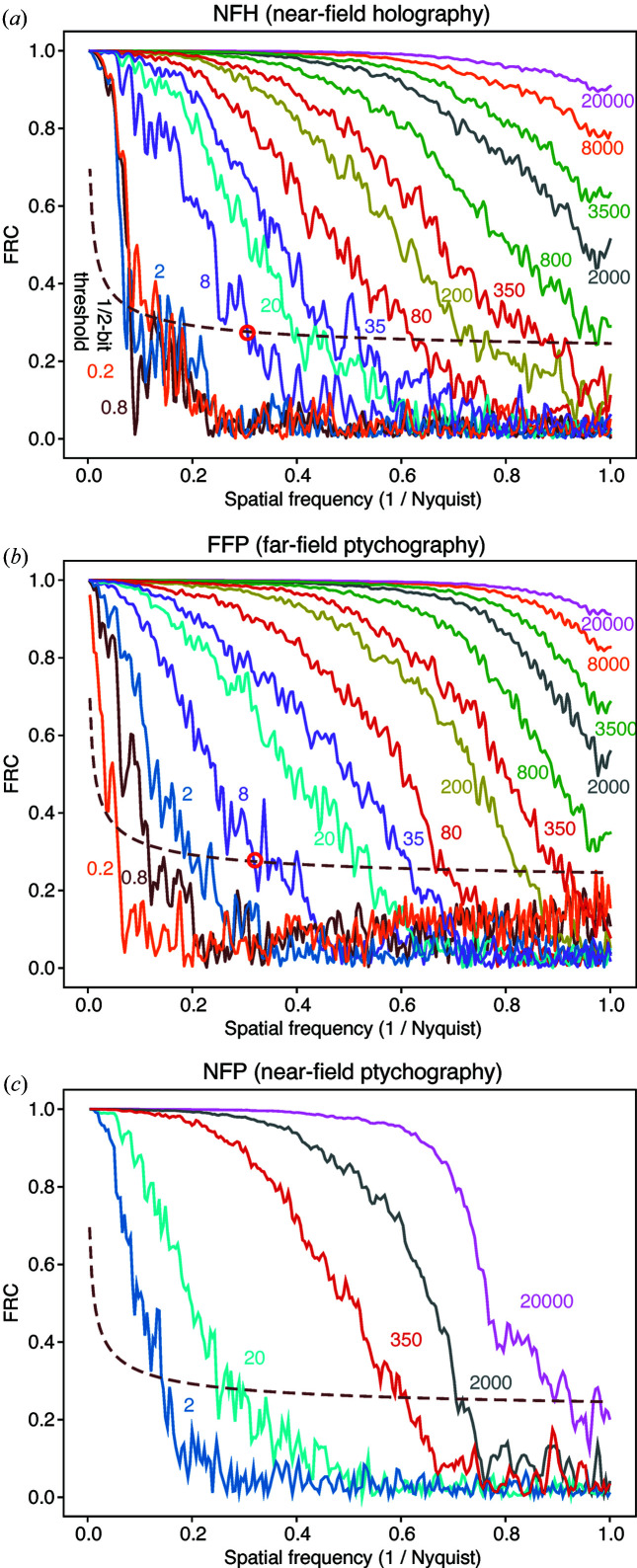
Fourier ring correlation (FRC) curves for two images reconstructed from separate instances of Poisson-noise-included simulated data sets, for (*a*) NFH, (*b*) FFP and (*c*) NFP. Only results obtained using the LSQ cost function of equation (7) are shown. Each curve is labeled with the fluence 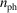 in quanta per pixel. Also shown on the plot is the 1/2-bit threshold curve that is commonly used to define the achieved spatial resolution based on the spatial frequency of the crossing with the experimental FRC curve (van Heel & Schatz, 2005 ▸), as indicated by red circles for a fluence of 8 in (*a*) and (*b*). These FRC crossing normalized spatial frequencies are used in Fig. 6 ▸.

**Figure 6:**
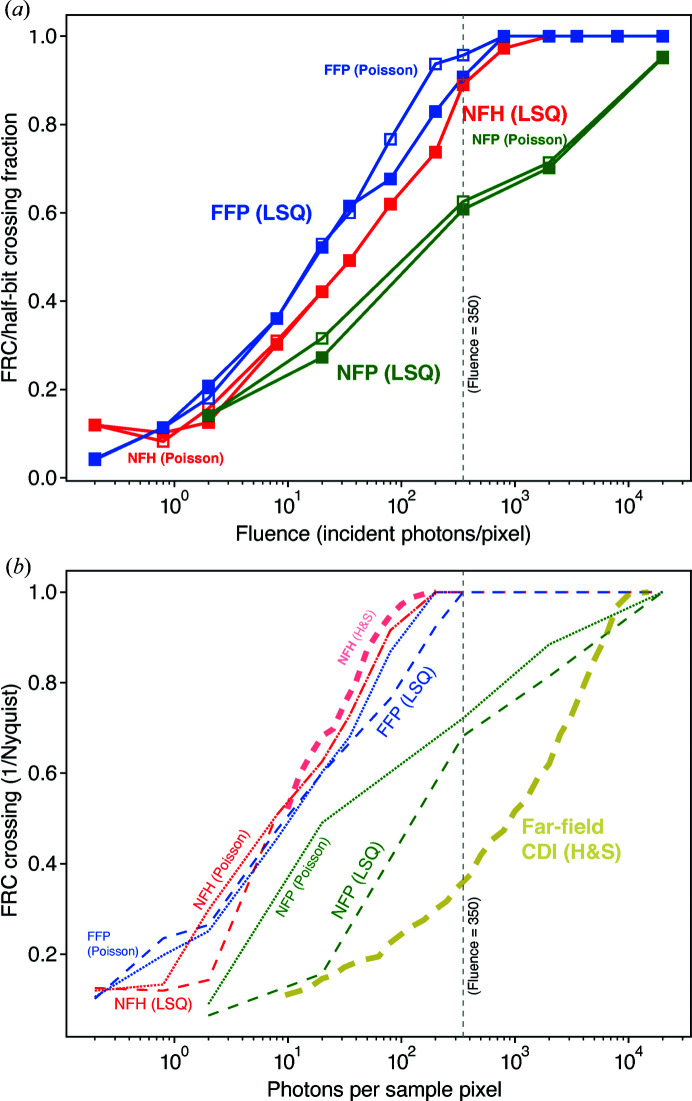
Values for the crossing between the FRC curves of Fig. 5 ▸ and the half-bit resolution criterion (van Heel & Schatz, 2005 ▸), shown as a fraction of the Nyquist spatial frequency limit of 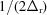. This is done for NFH, FFP and NFP. In (*a*), the crossing values are shown for the FRC analysis between reconstructed images obtained from two instances of Poisson noise, as normally required. The curves are not entirely smooth due to the sensitivity of the FRC crossing to the exact noise instance of the FRC curves shown in Fig. 5 ▸, but they show that one achieves full spatial resolution with NFH and FFP at fluences near the value of 350 quanta per pixel (shown with a vertical dashed line) estimated after equation (12). In prior work (Hagemann & Salditt, 2017 ▸), the FRC crossing analysis was done by comparison of one noise instance with the ‘ground-truth’ object of the cell phantom, so (*b*) shows our results for an equivalent ‘ground-truth’ FRC crossing as dashed lines. Also shown in (*b*) are the approximate results of the previous study (Hagemann & Salditt, 2017 ▸) of NFH, labeled with ‘(H&S)’ as obtained by tracing of the published figure. [The previous study plotted the FRC crossing as a function of 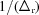, so we have multiplied the FRC crossing fractions by a factor of 2.] As can be seen, our ‘ground-truth analysis’ results and the ‘H&S’ results are reasonably consistent for the case of NFH. The previous study also considered far-field CDI [‘Far-field CDI (H&S)’], where the entire object array is illuminated and a finite-support constraint is applied during iterative reconstruction.

Another important metric for evaluating two separate instances of equally noisy images is to examine the correlation of their Fourier transforms as a function of radial spatial frequency 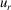, leading to the Fourier shell correlation for 3D images or the Fourier ring correlation (FRC) for 2D images (Saxton & Baumeister, 1982 ▸; van Heel, 1987 ▸) given by
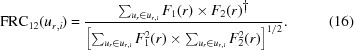
High-resolution low-noise images will show strong correlation at high spatial frequencies, while lower-resolution noisier images will show poorer correlation at high spatial frequencies. It is common to assign a spatial resolution value based on the crossing of the FRC with a half-bit threshold value (van Heel & Schatz, 2005 ▸). The resulting FRC analysis (plotted only for LSQ results) shown in Fig. 5 ▸ indicates that both NFH and FFP deliver full-resolution images at high photon fluences with similar information distribution over the spatial frequency below the Nyquist limit. On the other hand, NFP largely loses correlativity at mid-high frequency even at 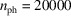 due to the uncorrelated artifacts. This figure also highlights the half-bit resolution FRC crossing point with a red circle for the case of an incident fluence of 8 quanta per pixel for each imaging method. This measure of the spatial resolution as a fraction of the 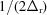 Nyquist spatial frequency is shown in Fig. 6 ▸(*a*), where one can see that both NFH and FFP approach full resolution at a fluence near the estimate of 350 quanta per pixel found using equation (12), while NFP barely reaches full resolution at 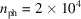
 photons per pixel. Because of the noise fluctuations present in the FRC curves, the FRC/half-bit crossing fraction may show some variations depending on the particular instances of data Poisson noise; this explains the non-smooth trend of the FRC crossing values shown in Fig. 6 ▸(*a*).

The fraction of the Nyquist limit spatial frequency shown in Fig. 6 ▸(*a*) was calculated by FRC analysis from two separate instances of Poisson noise at each fluence value and each imaging mode. However, a prior study has carried out FRC analysis by comparing a noisy image against the ground-truth image of the noise-free cell phantom (Hagemann & Salditt, 2017 ▸). We have therefore calculated this ‘ground-truth’ FRC crossing value, as well as tracing the curves shown in Fig. 4(*a*) of this previous analysis (Hagemann & Salditt, 2017 ▸) for both NFH and far-field CDI (where the latter involves a single diffraction pattern from illuminating the entire object array, and the use of a finite support in iterative phase retrieval). We show in Fig. 6 ▸(*b*) up to two FRC/half-bit crossing curves for each experiment/cost function type: the crossing obtained by comparing one low-fluence image with the ground-truth image (for NFH, FFP and NFP), and the traced values from Fig. 4(*a*) of the previous analysis (Hagemann & Salditt, 2017 ▸) (for NFH and CDI). As can be seen, there is reasonable agreement betwen our FRC crossing results and those of the previous analysis (Hagemann & Salditt, 2017 ▸) for the case of NFH with a ground-truth reference, even though the previous analysis used a slightly different reconstruction algorithm (the RAAR algorithm; Luke, 2005 ▸). In addition, FFP, NFH and NFP all show improved performance relative to far-field CDI, which suffers from well known difficulties (Miao *et al.*, 2005 ▸; Thibault *et al.*, 2006 ▸; Williams *et al.*, 2007 ▸; Huang *et al.*, 2010 ▸).

Overall, the above analyses and discussions suggest similar performance between FFP and NFH over a wide range of fluence, although FFP performs slightly better in terms of SNR (especially at low photon fluence). However, it should also be noted that FFP has certain extra requirements: it requires a high degree of coherence over the entire beam used, while NFH requires high coherence within the region of Fresnel fringes from a feature in a specimen but not over the entire illumination field. (As an example, with Fresnel fringes extending to 20 µm, one could use a 200 µm-wide beam with 20 µm coherence width to image a larger FOV with higher flux if using a partially coherent source.) FFP also requires accurate movement of a probe beam relative to the sample (though computational probe position refinement can also help correct for errors; Guizar-Sicairos & Fienup, 2008 ▸; Zhang *et al.*, 2013 ▸). Additionally, all our FFP results shown above were reconstructed with a known probe function. In reality, it is often the case that the probe needs to be reconstructed along with the object, which is straightforward (Thibault *et al.*, 2008 ▸, 2009 ▸) but which also requires additional computation. Finally, our results show poorer performance for NFP relative to FFP and NFH, but this may be due in part to the fact that we employed a finite-support constraint to suppress the twin image in NFH, but not in NFP (nor did we use a finite-support constraint in FFP, since the limited spatial extent of the probe function acts in some ways as a per-probe-position finite-support constraint). The use of a finite-support constraint helps tremendously with reconstruction fidelity in NFH, and one could expect that it would improve the performance of NFP as well for those specimens that do fit within a finite-support region.

## 4. Related literature   

The following additional references are cited in the supporting information: Huang *et al.* (2014 ▸), Maiden & Rodenburg (2009 ▸).

## 5. Conclusion   

We have considered a variety of coherent imaging methods and how they can perform with varying X-ray fluence. While the brightness (and thus coherent flux) of synchrotron light sources has been increasing dramatically (with the next advance being provided by diffraction-limited storage rings; Eriksson *et al.*, 2014 ▸), radiation dose sets a limit to achievable resolution (Sayre *et al.*, 1977*b* ▸; Howells *et al.*, 2009 ▸; Du & Jacobsen, 2018 ▸). Therefore it is usually desirable to use the lowest fluence possible, and instead use increasing coherent flux to image larger FOV with shorter exposure times, or a greater number of specimens to give better statistical sampling of a phenomenon.

We have used the same AD-based optimization method for image reconstruction to compare the performance of NFH, FFP and NFP at low specimen fluence values. Though this reconstruction algorithm is slightly different from what was used in a previous study (Hagemann & Salditt, 2017 ▸) that compared NFH with single-exposure far-field CDI, we have obtained quite similar results for NFH as shown in Fig. 6 ▸(*b*), as well as in a comparison of our Fig. 3 ▸(*b*) with Fig. 4(*c*) of the previous study. The previous study showed that NFH gives greatly superior results compared with far-field CDI, but far-field CDI is known to be very challenging due to the experimental difficulty of obtaining an object that has truly zero scattering outside a defined region (the finite support), and due to the sensitivity of the reconstruction to the correct ‘tightness’ of the support and the accuracy of recording the strong low-spatial-frequency diffraction signal (Miao *et al.*, 2005 ▸; Thibault *et al.*, 2006 ▸; Williams *et al.*, 2007 ▸; Huang *et al.*, 2010 ▸). FFP removes the requirement for the object to be within a finite-support constraint, and if a lens focus is used to provide the scanned coherent illumination spot the spreading of the signal in the far-field diffraction pattern helps reduce the dynamic range demands placed on the detector (Thibault *et al.*, 2008 ▸). In addition, the partitioning of data recording into a set of distinct regions of the object may provide some additional information beyond what one obtains when illuminating the entire object in one exposure, which may be why we observe slightly improved SNR from FFP relative to NFH in this computational study.

We conclude that the imaging method used does play some role in the quality of an image that one can obtain from a given fluence on the specimen. [We also note that, if an optic were to be used to record a direct image with no reconstruction algorithm required, one would need to increase the fluence to account for the focusing efficiency of the optic (Huang *et al.*, 2009 ▸), which is often below 20% for the case of Fresnel zone plates used for X-ray microscopy (Kirz, 1974 ▸).] However, it is still photon fluence that dominates the achievable reconstruction, as has long been suggested on the basis of theoretical analyses (Glaeser, 1975 ▸; Sayre *et al.*, 1977*b* ▸; Howells *et al.*, 2009 ▸; Du & Jacobsen, 2018 ▸) and simulation studies (Huang *et al.*, 2009 ▸; Jahn *et al.*, 2017 ▸). While previous studies using NFH suggested that one could obtain images at reduced radiation dose compared with far-field imaging methods (Bartels *et al.*, 2015 ▸), they did not include a systematic analysis of resolution versus fluence. Such an analysis was included in a prior computational study (Hagemann & Salditt, 2017 ▸), but it compared NFH with far-field CDI, rather than with a more robust far-field method like FFP. When CDI in this comparison is replaced with FFP, we start to observe that the two techniques provide similar spatial resolution at a wide range of photon fluence, as indicated by our FRC analysis. By bringing NFP into the comparison, we can state with more confidence that near-field and far-field imaging are generally equivalent in the resolution that they can achieve, because information redundancy due to a scanning-type acquisition scheme does not necessarily provide an advantage, and thus does not really compensate for the resolution of FFP. We therefore conclude that the sample can be near or far; wherever you are, photon fluence on the specimen sets a fundamental limit to spatial resolution.

## Supplementary Material

Supporting information file. DOI: 10.1107/S1600576720005816/dy5001sup1.pdf
