# Supplementary material for: Near, far, wherever you are: simulations on the dose efficiency of holographic and ptychographic coherent imaging
Source: J Appl Crystallogr. 2020 May 27;53(Pt 3):748–59. doi: 10.1107/S1600576720005816 (PMC7312132; doi:10.1107/S1600576720005816)
Supplement: Supplementary file 1 [file j-53-00748-sup1.pdf]

JOURNAL OF
APPLIED
CRYSTALLOGRAPHY

Volume 53 (2020)

Supporting information for article:

Near, far, wherever you are: simulations on the dose efficiency of holographic and ptychographic coherent imaging

This article will form part of a virtual special issue of the journal on ptychography software and technical developments.

Ming Du, Doğa Gürsoy and Chris Jacobsen

Near, far, wherever you are: simulations on the dose efficiency of holographic and ptychographic coherent imaging

Supplementary Materials

Ming Du[a], Doğa Gürsoy[a,b], and Chris Jacobsen[a,c,d]

[a]Advanced Photon Source, Argonne National Laboratory, Argonne, IL 60439 USA
[b]Department of Electrical Engineering and Computer Science, Northwestern University, Evanston, IL 60208 USA
[c]Department of Physics and Astronomy, Northwestern University, Evanston, IL 60208 USA
[d]Chemistry of Life Processes Institute, Northwestern University, Evanston, IL 60208 USA

1 Influence of probe spacing in far-field ptychography reconstruction

When a probe scan grid that is too coarse is used for far-field ptychography (FFP), the number of photons in the "tails" of the probe function becomes low enough that there can be insufficient overlap between probe positions compared to the high-photon-number case. In this case, a grid-like artifact will appear in the reconstructed image [1, 2], especially when using the least-square (LSQ) cost function. The scan grid of 68×66 probe positions we used to generated all reconstructions shown in the main text is fine enough to suppress this artifact at low fluence, but when probe spacing is doubled, the artifacts become obvious. We demonstrate this effect in Fig. S1, which includes far-field ptychography (FFP) reconstructions of the dataset generated with a photon fluence of 20 photons per reconstruction array pixel, using both a fine (68×66 probe positions) and coarse (34×33 probe positions) probe spacing grid. Both grids covers the same area indicated by the yellow dashed box, so the coarser grid has twice the probe spacing in the horizontal and vertical directions. To keep the number of photons per reconstruction array pixel equal (and thus the fluence – and the dose to the specimen – equal), we supplied 1/4 the number of photons per probe position in the fine grid relative to the coarse grid. With the same per-pixel photon dose, the coarse grid reconstruction using the LSQ cost function, which is used by popular algorithms like ePIE [3], shows obvious grid artifacts. This grid artifact can produce correlations based on artifacts rather than specimen features when comparing two independent reconstructions in Fourier shell correlation or FSC resolution analysis, and thus can provide a biased measure of higher-than-justified resolution

at low exposures.

When using the Poisson cost function, grid artifacts are greatly reduced, and the features in the image become sharper. At the same time, however, another type of artifact (fringes around fine features, as discussed in the main article text) emerges in Poisson reconstructions.

References

[1] Oliver Bunk, Martin Dierolf, Søren Kynde, Ian Johnson, Othmar Marti, and F Pfeiffer. Influence of the overlap parameter on the convergence of the ptychographical iterative engine. Ultramicroscopy, 108(5):481–487, April 2008.

[2] Xiaojing Huang, Hanfei Yan, Ross J Harder, Yeukuang Hwu, Ian K Robinson, and Yong S Chu. Optimization of overlap uniformness for ptychography. Optics Express, 22(10):12634–12644, 2014.

[3] Andrew M Maiden and John M Rodenburg. An improved ptychographical phase retrieval algorithm for diffractive imaging. Ultramicroscopy, 109(10):1256–1262, August 2009.

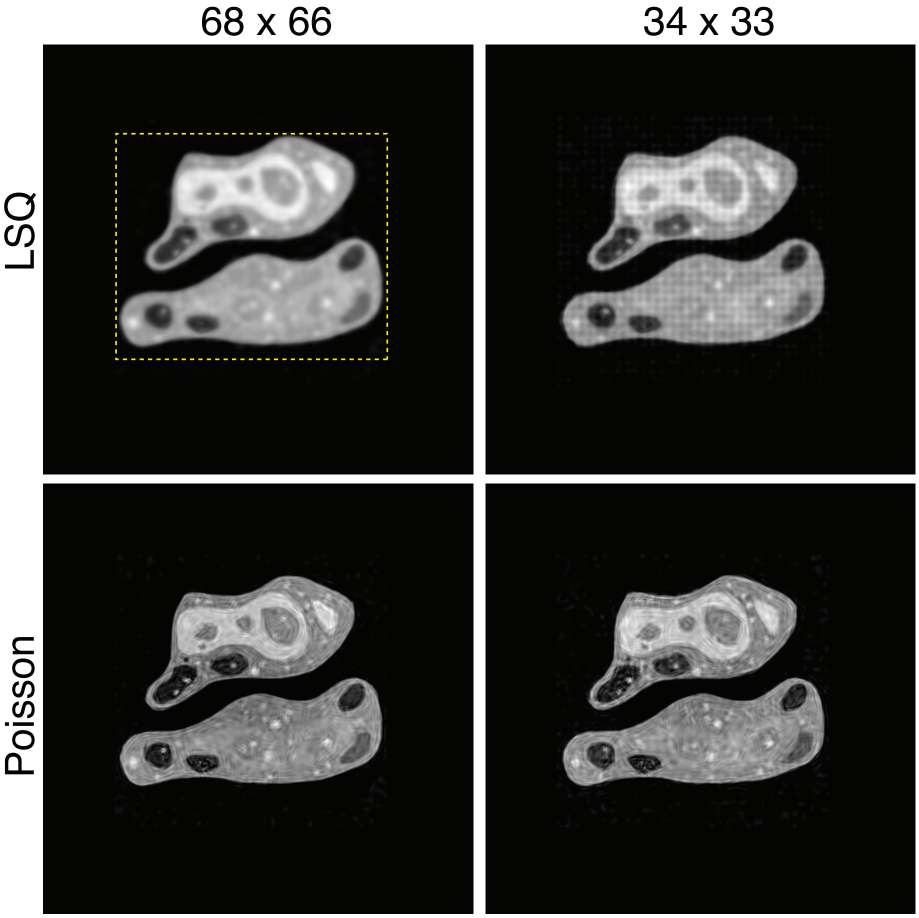

Figure S1: Reconstructions of far-field ptychography (FFP) data with a photon fluence of 20 photons per reconstruction array pixel using a fine (68 × 66 probe positions) and a coarse (34 × 33 probe positions) scan grid, respectively. Reconstructions obtained using the least-squares (LSQ) and the Poisson cost function are shown for each scan grid. Both grids cover the same area relative to the sample, as shown by the yellow dashed box.
